# Predicting IDH genotype in gliomas using FET PET radiomics

**DOI:** 10.1038/s41598-018-31806-7

**Published:** 2018-09-06

**Authors:** Philipp Lohmann, Christoph Lerche, Elena K. Bauer, Jan Steger, Gabriele Stoffels, Tobias Blau, Veronika Dunkl, Martin Kocher, Shivakumar Viswanathan, Christian P. Filss, Carina Stegmayr, Maximillian I. Ruge, Bernd Neumaier, Nadim J. Shah, Gereon R. Fink, Karl-Josef Langen, Norbert Galldiks

**Affiliations:** 1 Inst. of Neuroscience and Medicine (INM-3, −4, −5), Forschungszentrum Juelich, Juelich, Germany; 2 Dept. of Stereotaxy and Functional Neurosurgery, University of Cologne, Cologne, Germany; 3 Dept. of Neurology, University of Cologne, Cologne, Germany; 4 Dept. of Neuropathology, University of Cologne, Cologne, Germany; 5 Dept. of Neurology, University Hospital RWTH Aachen, Aachen, Germany; 6 Dept. of Nuclear Medicine, University Hospital RWTH Aachen, Aachen, Germany; 7 Center of Integrated Oncology (CIO), Universities of Cologne and Bonn, Cologne, Germany

## Abstract

Mutations in the isocitrate dehydrogenase (IDH mut) gene have gained paramount importance for the prognosis of glioma patients. To date, reliable techniques for a preoperative evaluation of IDH genotype remain scarce. Therefore, we investigated the potential of O-(2-[^18^F]fluoroethyl)-L-tyrosine (FET) PET radiomics using textural features combined with static and dynamic parameters of FET uptake for noninvasive prediction of IDH genotype. Prior to surgery, 84 patients with newly diagnosed and untreated gliomas underwent FET PET using a standard scanner (15 of 56 patients with IDH mut) or a dedicated high-resolution hybrid PET/MR scanner (11 of 28 patients with IDH mut). Static, dynamic and textural parameters of FET uptake in the tumor area were evaluated. Diagnostic accuracy of the parameters was evaluated using the neuropathological result as reference. Additionally, FET PET and textural parameters were combined to further increase the diagnostic accuracy. The resulting models were validated using cross-validation. Independent of scanner type, the combination of standard PET parameters with textural features increased significantly diagnostic accuracy. The highest diagnostic accuracy of 93% for prediction of IDH genotype was achieved with the hybrid PET/MR scanner. Our findings suggest that the combination of conventional FET PET parameters with textural features provides important diagnostic information for the non-invasive prediction of the IDH genotype.

## Introduction

Since the recent update of the World Health Organization (WHO) Classification of Tumors of the Central Nervous System^1^, the revised classification now integrates histology and molecular features, particularly giving pivotal attention to the isocitrate dehydrogenase (IDH) mutation and 1p/19q co-deletion, which allow a prediction of the individual response to therapy^2–4^. Furthermore, the inclusion of the IDH mutation status enables an improved assessment of the individual prognosis, compared to the WHO grades defined by the 2007 classification^1,2,5–7^.

IDH mutations (mut) are frequently observed in lower-grade gliomas (WHO grades II and III) and secondary glioblastomas (GBM)^8^. In contrast, IDH mutations in primary GBM are rare^8^. From a clinical point of view, most IDH wild type (wt) lower-grade gliomas are comparable to GBM^7,8^. Furthermore, GBM patients with IDH mutations have a significantly longer overall survival compared to their IDH wt counterparts (31 months vs. 15 months)^7^. Moreover, current guidelines recommend that treatment decisions should be based on the IDH status and 1p/19q co-deletion^9^. However, to date tissue samples are inevitable if one wants to assess the IDH status. Hence, a reliable method for the non-invasive assessment of the IDH genotype is of great interest. Magnetic resonance spectroscopy has already been used for the detection of 2-hydroxyglutarate (2-HG), a specific tumor metabolite present in IDH mut gliomas^10^. However, measurements of 2-HG in clinical routine are challenging due to the very small and complex signals of the metabolite^11,12^.

**Figure 1:**
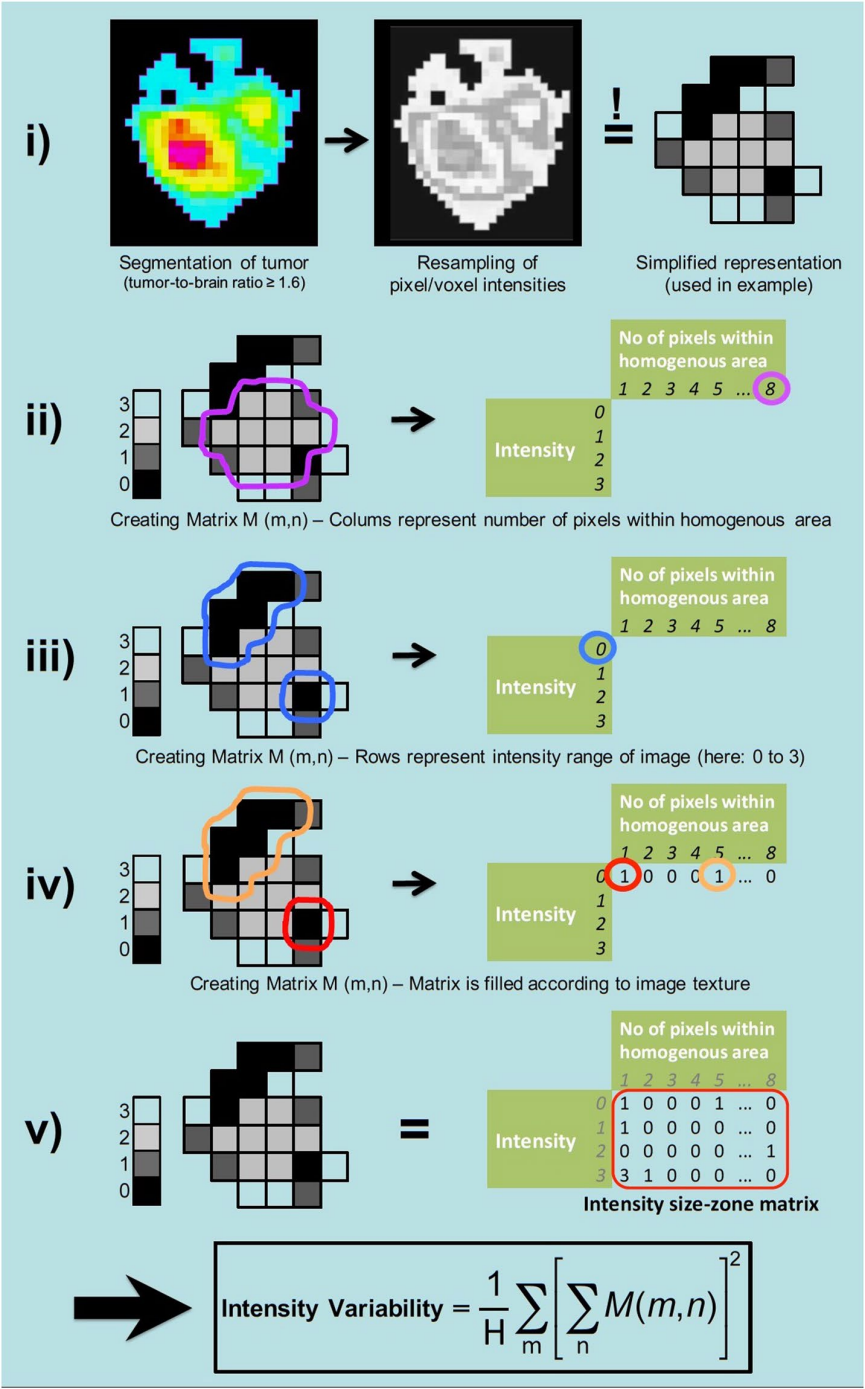
What are textural features? In this example, the basic processing steps and the principle of textural feature analysis is illustrated. The feature *Intensity Variability* used in the example describes areas of similar intensity and is a measure of image (or tumor) heterogeneity; equivalent to the parameter *Grey-Level Non-Uniformity for zone* (GLNUz) used in the manuscript. Index H represents the number of homogenous zones in the volume of interest.

Currently, textural feature analysis in the context of radiomics increasingly gain attraction in Oncology and Neuro-Oncology and has already been applied for the identification of the IDH mutational status in patients with WHO grade II gliomas according to fluid attenuated inversion recovery (FLAIR) MR images^13^ as well as in patients with WHO grade II and III gliomas based on MR diffusion tensor imaging^14^. Furthermore, textural feature analysis of O-(2-[^18^F]fluoroethyl)-L-tyrosine (FET) PET images has been used for glioma grading^15^, diagnosis of pseudoprogression in high-grade gliomas^16^, and the differentiation of radiation-induced changes from brain metastasis recurrence^17^. Figure 1 illustrates the basic fundamental processing steps and the general concept of textural feature analysis.

Goal of our study was the evaluation of FET PET textural features compared to and in combination with static and dynamic FET PET parameters for the preoperative differentiation of IDH mut from IDH wt gliomas.

## Results

**Figure 2:**
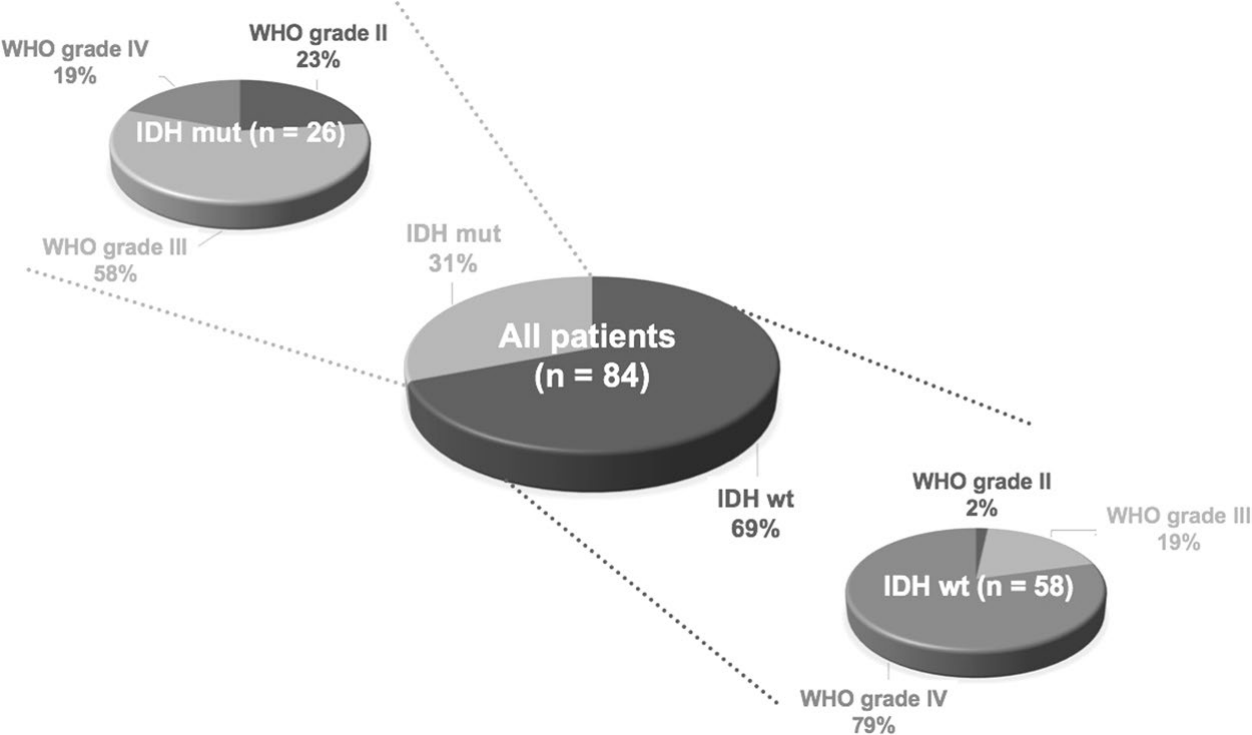
Patient cohort and distribution of IDH genotypes and WHO grades.

Based on the results from the neuropathological assessment of the IDH status, 26 gliomas (31%) were IDH mut and 58 (69%) were IDH wt. From the 56 patients scanned on the stand-alone PET scanner, 15 (27%) had IDH mut gliomas and the remaining 41 (73%) patients had an IDH wt. From the 28 patients measured on the hybrid PET/MR scanner, 11 (39%) had IDH mut gliomas and the remaining 17 (61%) patients had an IDH wt. Further details are provided in Table 1 and Fig. 2. A summary of the conventional FET PET parameters for all three patient groups is provided in Table 2.

**Table 1:** Demographic and clinical data of all patients.

Patient cohort	All	Subgroup I (Stand-alone PET)	Subgroup II (Hybrid PET/MR)
Patients	84	56	28
Gender, f/m	34/50	24/32	10/18
Mean age ± SD	54 ± 14 y	55 ± 13 y	50 ± 16 y
Age range	22–76 y	23–76 y	22–76 y
IDH genotype, wt/mut	58/26	41/15	17/11
WHO grade II (wt/mut)	7 (1/6)	5 (0/5)	2 (1/1)
WHO grade III (wt/mut)	26 (11/15)	17 (10/7)	9 (1/8)
WHO grade IV (wt/mut)	51 (46/5)	34 (31/3)	17 (15/2)

**Table 2:** Summary of conventional FET PET parameters.

Patient cohort	All			Subgroup I (Stand-alone PET)			Subgroup II (Hybrid PET/MR)		
	IDH wt	IDH mut	p	IDH wt	IDH mut	p	IDH wt	IDH mut	p
TBR_mean_ ± SD	2.2 ± 0.3	2.1 ± 0.5	0.25	2.2 ± 0.3	2.3 ± 0.5	0.02	2.3 ± 0.5	1.9 ± 0.2	0.13
TBR_max_ ± SD	4.1 ± 1.2	3.9 ± 1.3	0.43	4.1 ± 1.1	4.3 ± 1.4	0.35	4.2 ± 1.4	3.3 ± 1.0	0.41
Slope [SUV/h] ± SD	−0.3 ± 0.5	0.3 ± 0.4	0.40	−0.2 ± 0.5	0.2 ± 0.5	0.55	−0.4 ± 0.6	0.3 ± 0.3	0.01
TTP [min]	28.2 ± 10.3	37.3 ± 7.4	0.01	28.0 ± 9.6	39.2 ± 7.7	0.40	28.7 ± 12.2	34.8 ± 6.5	<0.01

TBR: tumor-to brain ratio; TTP: time to peak.

## Complete Patient Cohort (84 Patients)

### Prediction of the IDH status based on FET PET standard parameters

The parameter slope (slope of linear regression line evaluated 20–50 min post-injection) had the highest accuracy to predict the IDH genotype (accuracy, 80%; AUC, 0.79 ± 0.05; sensitivity, 58%; specificity, 90%; p < 0.01). The other standard parameters time-to-peak (TTP; time in minutes from the beginning of the dynamic acquisition up to the maximum SUV of the lesion), mean tumor-to-brain ratio (TBR_mean_) and maximum tumor-to-brain ratio (TBR_max_) lead to diagnostic accuracies within the range of 71–73%. A summary of the results is provided in Supplementary Table 1.

### Prediction of the IDH status based on FET PET textural features

The histogram-based parameter skewness (SkewnessH) and the parameter long run high grey-level emphasis (LRHGE) had the highest diagnostic accuracy for predicting IDH genotype (SkewnessH: accuracy, 71%; AUC, 0.53 ± 0.07; sensitivity, 31%; specificity, 90%; p = 0.66; LRHGE: accuracy, 71%; AUC, 0.52 ± 0.07; sensitivity, 8%; specificity, 100%; p = 0.75). The diagnostic accuracies of the other textural features were within the range of 52–70%. A summary of the results is provided in Supplementary Table 1.

### Prediction of the IDH status based on combination of parameters

**Figure 3:**
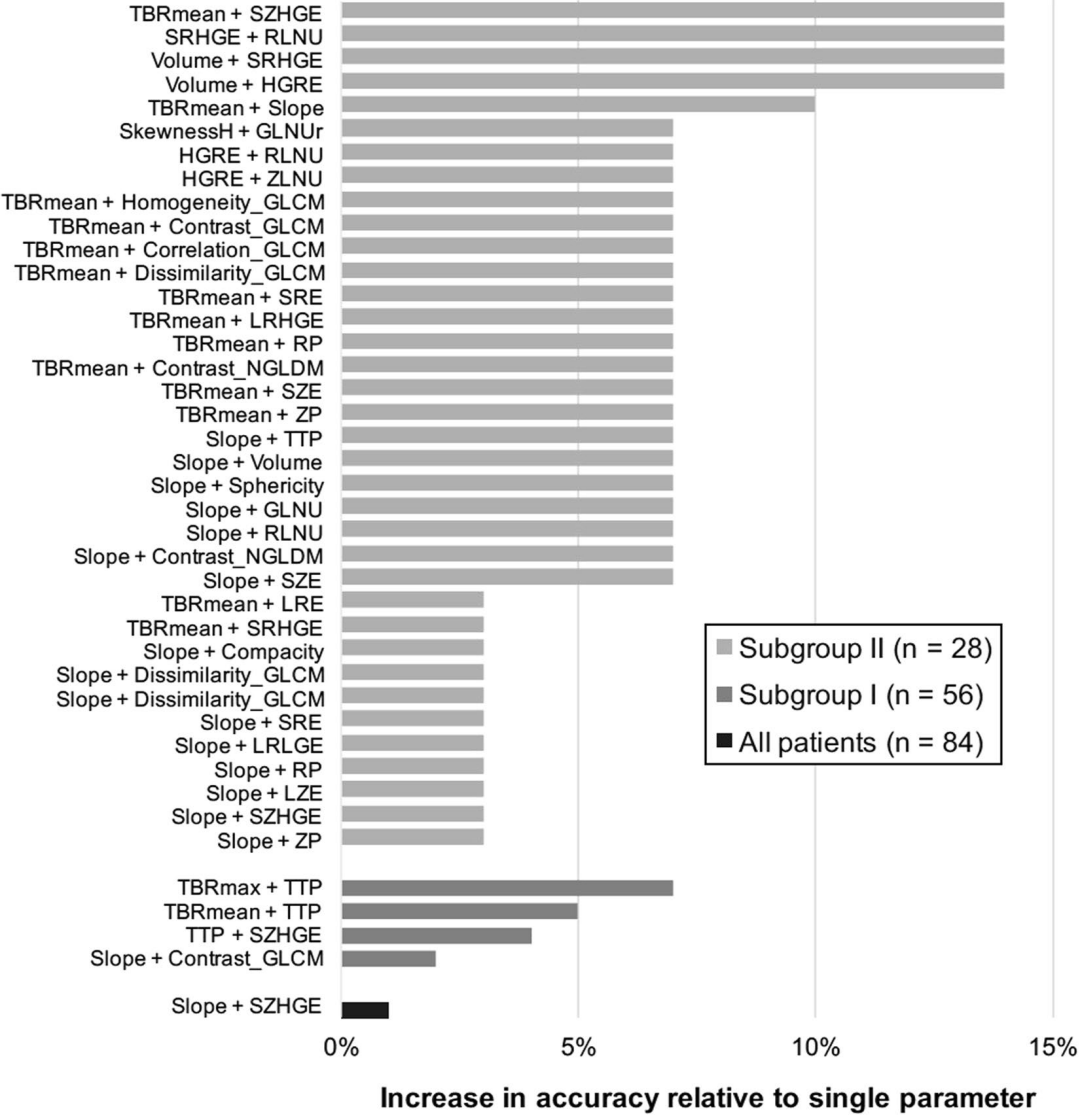
Increase in diagnostic accuracy to predict IDH genotype after combination of parameters (relative difference to accuracy of respective single parameter). GLCM: Grey-level co-occurrence matrix; GLNUr: Grey-level non-uniformity for run; HGRE: High grey-level run emphasis; LRE: Long-run emphasis; LRHGE: Long-run high grey-level emphasis; LRLGE: Long-run low grey-level emphasis; LZE: Long-zone emphasis; NGLDM: Neighborhood grey-level different matrix; RLNU: Run length non-uniformity; RP: Run percentage; SkewnessH: Skewness of histogram; SRE: Short-run emphasis; SRHGE: Short-run high grey-level emphasis; SZE: Short-zone emphasis; SZHGE: Short-zone high grey-level emphasis; TBR: tumor-to-brain ratio; TTP: time to peak; ZLNU: Zone length non-uniformity; ZP: Zone percentage.

The combination of the standard parameter slope with the textural feature short zone high grey-level emphasis (SZHGE) slightly increased the diagnostic accuracy to 81% (sensitivity, 54%; specificity, 93%; p < 0.01). The overall accuracy of the model validation was 79% after 5-fold cross-validation and 80% after 10-fold cross-validation. Further details on the model performance and the validation are provided in Table 3. Other combinations did not further increase the diagnostic accuracy. The results are summarized in Supplementary Table 4 and Fig. 3.

**Table 3:** Results of best parameter combinations.

Patient cohort	Parameter 1	Parameter 2	Accuracy no validation	Accuracy 5-fold CV	Accuracy 10-fold CV	p (Bonferroni)
Complete	Slope [SUV/h]	SZHGE	0.81	0.79	0.80	<0.01
(n = 84)						
Subgroup I	TTP [min]	SZHGE	0.84	0.82	0.80	0.10
(n = 56)						
Subgroup II	TBR_mean_	SZHGE	0.93	0.82	0.86	<0.01
(n = 28)						

CV: cross-validation; SZHGE: Short-zone high grey-level emphasis; TBR: tumor-to brain ratio; TTP: time to peak.

## Subgroup I – 56 Patients Examined on the Stand-Alone PET Scanner

### Prediction of the IDH status based on FET PET standard parameters

The parameters slope and TTP had the highest diagnostic accuracy to predict the IDH genotype (slope: accuracy, 80%; AUC, 0.74 ± 0.07; sensitivity, 53%; specificity, 90%; p = 0.01; TTP: accuracy, 80%; AUC, 0.80 ± 0.06; sensitivity, 40%; specificity, 95%; p < 0.01). The diagnostic accuracies of the other standard parameters TBR_mean_ and TBR_max_ were 77% and 75%, respectively. A summary of the results is provided in Supplementary Table 2.

### Prediction of the IDH status based on FET PET textural features

All of the best sixteen textural parameters yielded a diagnostic accuracy of 75%. The diagnostic accuracies of the other textural features were lower and within the range of 63–73%. A summary of the results is provided in Supplementary Table 2.

### Prediction of the IDH status based on combination of parameters

The largest increase in accuracy to predict the IDH genotype was achieved by combining the standard parameter TTP with the textural feature SZHGE (accuracy, 84%; sensitivity, 53%; specificity, 95%; p = 0.10). The overall accuracy of the model validation was 82% after 5-fold cross-validation and 80% after 10-fold cross-validation. Further details on the model performance and the validation are provided in Table 3. Combinations of TBR_max_ with TTP, TBR_mean_ with TTP, and slope with Contrast_GLCM, respectively, increased the accuracy to 82%. Other combinations did not further increase the accuracy. The results are summarized in Supplementary Table 4 and Fig. 3.

## Subgroup II – 28 Patients Scanned on the Hybrid PET/MR Scanner

### Prediction of the IDH status based on FET PET standard parameters

The parameters TBR_mean_ and slope had the highest accuracy to predict the IDH genotype (TBR_mean_: accuracy, 79%; AUC, 0.84 ± 0.07; sensitivity, 91%; specificity, 71%; p < 0.01; slope: accuracy, 79%; AUC, 0.85 ± 0.07; sensitivity, 73%; specificity, 82%; p < 0.01). The diagnostic accuracies of the other standard parameters TBR_max_ and TTP were 75% and 64%, respectively. A summary of the results is provided in Supplementary Table 3.

### Prediction of the IDH status based on FET PET textural features

The histogram-based parameter kurtosis (KurtosisH) had the highest diagnostic accuracy to predict the IDH genotype (accuracy, 79%; AUC, 0.78 ± 0.09; sensitivity, 82%; specificity, 76%; p = 0.01). The diagnostic accuracies of the other textural features were lower and within a range of 57–75%. A summary of the results is provided in Supplementary Table 3.

### Prediction of the IDH status based on combination of parameters

The largest increase in accuracy was achieved by combining TBR_mean_ with the textural feature SZHGE (accuracy, 93%; sensitivity, 91%; specificity, 94%; p < 0.01). The overall accuracy of the model validation was 82% after 5-fold cross-validation and 86% after 10-fold cross-validation. Further details on the model performance and the validation are provided in Table 3. Thirty-five other parameter combinations resulted in an increased diagnostic accuracy relative to the respective single parameter within the range of 82–89%. The results are summarized in Table 3, Supplementary Table 4 and Fig. 3.

## Discussion

In the context of radiomics, the aim of this study was to evaluate the diagnostic potential of textural feature analysis of tumoral FET uptake to predict the IDH genotype of glioma patients. Radiomics is a term that is used to describe the application of computational methods to extract a large number of parameters from medical imaging data to improve the diagnostic, prognostic and predictive accuracy.

Textural feature analysis is one tool within the concept of radiomics that objectively and quantitatively describes particularly structural heterogeneity. The calculated textural features are abstract measures that mathematically describe the intensity variations of the underlying image beyond visual perception. It has been demonstrated that the combination of different methods of image analysis (i.e., static and dynamic FET PET parameters in combination with textural features) yields additional information about tumor biology and thus, allows the prediction of the IDH genotype in glioma patients with high accuracy.

Various types of extracranial tumors have been already investigated using textural feature analysis such as non-small cell lung cancer^18^, cervical cancer^19^, or nasopharyngeal carcinoma^20^ based on [^18^F]-2-fluoro-2-deoxy-D-glucose (FDG) PET images. Furthermore, the usefulness of textural feature analysis of conventional MRI images to predict molecular markers in patients with brain tumors has been demonstrated. For example, Korfiatis and colleagues showed the ability of textural features to predict the O^6^-methylguanine-DNA methyltransferase (MGMT) methylation status in GBM patients on T2-weighted MRI with a high accuracy (AUC, 0.85)^21^. Furthermore, the IDH genotype was evaluated using textural feature analysis of MRI data. Zhang and co-workers investigated textural feature analysis of preoperative MRI of high-grade glioma patients to predict the IDH status. They achieved a diagnostic accuracy of 89%^22^. Similar results were obtained in a recent study using deep learning-based radiomics to predict the IDH genotype in WHO grade II gliomas based on MRI with a diagnostic accuracy ranging between 85% and 95%^23^. Another study from Eichinger and colleagues^14^ generated local binary pattern textural features from MR diffusion tensor imaging data of 79 untreated WHO grade II and III glioma patients. They achieved a high diagnostic accuracy of 95% in an independent validation set. However, the studies mentioned above used highly pre-selected patient cohorts which require a priori knowledge of tumor grade from histological samples which contradicts the additional benefit of a non-invasive method for assessment of the IDH genotype. On the contrary, our approach was tested and can be applied on a more realistic mixed patient population without prior preselection. Additionally, MR-based methods require manual delineation of areas of contrast enhancement which limits the applicability of these approaches to tumors with a disrupted blood-brain barrier. Since the uptake of FET is independent of the blood-brain barrier integrity, FET PET radiomics can also be applied to non-contrast enhancing lesions.

Regarding PET imaging, the usefulness of FET PET textural feature analysis has been shown for glioma grading^15^, diagnosis of pseudoprogression^16^, and the differentiation between recurrent brain metastasis and radiation-induced changes^17^. To the best of our knowledge, this study is the first to assess the potential of textural feature analysis of FET PET for the non-invasive prediction of the IDH genotype in gliomas. Our data thus complement and extend equivalent radiomics-based approaches using MRI data.

We observed in the present study that independent of scanner type, diagnostic accuracies of the dynamic parameter slope and the static FET PET parameter TBR_mean_ were comparable (range, 73–80%). In all three patient groups, three textural features showed an accuracy of more than 70% (range, 70–79%). Interestingly, the combination of standard parameters with the textural feature SZHGE, which represents the distribution of short homogenous zones with high levels of intensity, increased the diagnostic accuracy independent of the PET scanner used. The diagnostic accuracy could be increased to 81% for the complete patient cohort and to 84% for subgroup I (stand-alone PET scanner). The highest diagnostic accuracy of 93% was observed for subgroup II (hybrid PET/MR scanner) after combining TBR_mean_ with SZHGE. These results suggest an influence of the spatial resolution of the scanner on textural feature analysis. The BrainPET scanner, which is integrated in the hybrid PET/MR system, is especially designed for brain imaging and offers PET images with a higher spatial resolution (center spatial resolution, approx. 3 mm full-width at half maximum (FWHM)) compared to standard stand-alone PET scanners such as the ECAT HR + (center spatial resolution, approx. 6 mm FWHM)^24,25^. Thus, in combination with the smaller reconstructed voxel size, PET images from the BrainPET scanner potentially encode more information about tumor heterogeneity. This might be an explanation for the highest diagnostic accuracy after combining standard PET parameters with textural features in patients measured with the hybrid PET/MR scanner (subgroup II).

As described previously^17^, in order to get reliable results from textural feature analysis of medical imaging data, several things have to be considered carefully.

Image quality is the parameter that has the biggest influence on the results of textural feature analysis. Especially in PET, the image quality depends on numerous parameters such as the applied reconstruction algorithm, the method of attenuation correction, the amount of injected radioactivity, the scan duration and the spatial resolution of the PET scanner. Comparative studies evaluating the effect of these parameters on textural feature analysis are necessary and a standardization is required to compare textural parameters from different studies. Until then, the applicability of textural feature analysis of PET images in multicenter studies is limited. Furthermore, the interpretation of the results from textural analyses, i.e. the link between the radiomics parameter and a physiological or pathophysiological meaning is restricted by the complex mathematical nature of the parameters. Only a few textural features such as contrast, can be easily linked to a visual aspect in the image. Unfortunately, most textural features, especially higher-dimensional ones can hardly be interpreted by means of human perception.

Textural feature analysis may thus prove useful as part of the diagnostic work-up rather than as a stand-alone measure. In order to assess the value of textural feature analysis in clinical routine, comparative studies evaluating the effect of different PET scanners and parameters on the comparability and reproducibility of textural feature analysis warrants further investigation^26–28^.

The use of hybrid PET/MR is steadily increasing in Neuro-Oncology and is also of great interest for future research in radiomics as textural parameters from different modalities allow to explore the benefit of multiparametric radiomic patterns. For example, Kickingereder and colleagues have recently demonstrated that a combination of three different MR contrasts leads to a radiomic signature that allows a non-invasive prediction and stratification of PFS and OS in 181 patients with IDH wt glioblastoma^29^. Additionally, textural feature analysis has a great potential also for treatment monitoring^30,31^, i.e., changes of textural parameters during treatment might be predictive to discriminate responders from non-responders at an early stage of therapy.

One limitation of our current study is its retrospective design. The robustness of the textural parameters of FET PET data for predicting IDH genotype needs to be confirmed in a larger prospective study. Also, the number of patients is relatively low and the number of parameters is high, which might potentially lead to overfitting of the data and an invalid model generation. To overcome this issue and generate a comprehensible and interpretable model, simple logistic regression was applied and the maximum number of parameters for model generation was restricted to two. Furthermore, 5-fold and 10-fold cross-validation was performed for model validation and Bonferroni post-hoc correction was applied. The different PET scanners used are another limitation. However, the utilized dataset represents a realistic clinical situation, in which a new method should prove its diagnostic value. To account for this, the patient cohorts were separately analyzed and the data from the two different PET scanners were used to investigate the influence of the image quality on the reproducibility of the standard and the textural parameters. Despite differences, all in all comparable results were obtained. Finally, from a practical viewpoint the post-processing of the image data is more complex than the evaluation of TBRs, which are commonly used in clinical routine. However, the post-processing takes less than thirty minutes and most of the steps could be automatized in order to speed-up the analyses.

In summary, the current study demonstrates that textural features in combination with standard FET PET parameters allow to non-invasively predict the IDH genotype of glioma patients with a high diagnostic accuracy.

## Patients and Methods

### Patients

From August 2007 to December 2016, 907 patients with suspected brain lesion were investigated using FET PET in the Institute of Neuroscience and Medicine, Forschungszentrum Juelich, Germany. Of those, 84 patients were included in this retrospective analysis (50 males, 34 females; mean age, 54 ± 14 years; range, 22–76 years) according to the following inclusion criteria: (i) newly diagnosed glioma, (ii) untreated prior to FET PET imaging, (iii) neuropathological diagnosis based on the WHO classification of 2016^1^ (patients with tumor classifications initially based on the WHO classification of 2007 were re-evaluated and re-classified according to the updated WHO classification of 2016), (iv) known IDH mutation status, (v) pathological FET uptake (TBR > 1.6), (vi) volume of pathological FET uptake >100 voxels^32^.

The patients’ neuropathological diagnoses after tumor resection (46% of patients) or biopsy (54% of patients) classified according to the WHO 2016 classification were 46 IDH wt GBM of the WHO grade IV; 5 IDH mut GBM of the WHO grade IV; 11 IDH wt anaplastic astrocytoma (AA) of the WHO grade III; 14 IDH mut AA of the WHO grade III; one IDH mut anaplastic oligodendroglioma (ODG) of the WHO grade III; one IDH wt astrocytoma of the WHO grade II; 3 IDH mut astrocytoma of the WHO grade II; and 3 IDH mut ODG of the WHO grade II. Further details of the patient cohort are presented in Table 1 and Fig. 2. All patients had provided written informed consent before each FET PET investigation. The ethics committee of the University Hospital RWTH Aachen approved the evaluation of retrospectively collected patient data. All methods were performed in accordance with the relevant guidelines and regulations.

### FET PET Imaging

The amino acid FET was produced and applied as described previously^33,34^. For each patient, a dynamic PET scan from 0 to 50 min post-injection (p.i.) of 3 MBq of FET per kg of body weight prior to histopathological confirmation was acquired. Fifty-six patients (31 IDH wt GBM (WHO grade IV), 3 IDH mut GBM (WHO grade IV), 10 IDH wt AA (WHO grade III), 7 IDH mut AA (WHO grade III), 2 IDH mut astrocytoma (WHO grade II), 3 IDH mut ODG (WHO grade II)) were measured on a stand-alone PET scanner (ECAT EXACT HR+, Siemens Medical Systems, Inc.) in 3D mode (32 rings; axial field of view, 15.5 cm; center spatial resolution, approx. 6 mm FWHM). The reconstructed dynamic dataset consisted of 16 time frames (5 × 1 min; 5 × 3 min; 6 × 5 min). A 10 min transmission scan using three rotating line sources (^68^Ge/^68^Ga) was used for attenuation correction. Before iterative OSEM reconstruction (16 subsets, 6 iterations), data were corrected for dead time, random and scattered coincidences.

Twenty-eight patients (15 IDH wt GBM (WHO grade IV), 2 IDH mut GBM (WHO grade IV), 1 IDH wt AA (WHO grade III), 7 IDH mut AA (WHO grade III), 1 IDH mut ODG (WHO grade III), 1 IDH wt astrocytoma (WHO grade II), 1 IDH mut astrocytoma (WHO grade II)), were scanned on a high-resolution 3 T hybrid PET/MR scanner (BrainPET, Siemens Medical Systems, Inc., 72 rings; axial field of view, 19.2 cm; center spatial resolution, approx. 3 mm FWHM). Image data were corrected for random and scatter coincidences, as well as dead time prior to OPOSEM reconstruction provided by the manufacturer (2 subsets, 32 iterations). The reconstructed dynamic data set consisted of 16 time frames (5 × 1 min; 5 × 3 min; 6 × 5 min). Since the hybrid PET/MR scanner does not provide a transmission source, attenuation correction was performed by a template-based approach using MRI^35^.

### IDH genotyping

After obtaining tumor tissue via open neurosurgery or stereotactic biopsy, all lesions were histologically classified according to the WHO classification 2016 of tumors of the central nervous system. For IDH mutation status, presence of an IDH1R132H protein expression was evaluated by immunohistochemistry. If immunostaining was negative, IDH was directly sequenced. The 1p/19q co-deletion status was analyzed by fluorescence *in situ* hybridization.

### Data Analysis

Summed PET images from 20–40 min p.i. were used for data analysis. Prior to further analyses, all images were motion-corrected using the PMOD software (Version 3.5.5, PMOD Technologies Ltd.). The FET uptake was normalized using the standardized uptake value (SUV) by dividing the radioactivity (kBq/mL) in the tissue by the radioactivity injected per gram of body weight. A spherical volume-of-interest (VOI) of constant size (diameter, 30 mm; volume, 14.2 mL) was placed in normal-appearing brain tissue including both grey and white matter contralateral to the lesion as reference^36^. The biological tumor volume was determined using a three-dimensional auto-contouring process using a TBR of 1.6 or more^37^. TBR_mean_ was calculated by dividing the mean SUV of the tumor VOI by the mean SUV of normal brain tissue. TBR_max_ was calculated by dividing the maximum SUV of the tumor VOI by the mean SUV of normal brain tissue.

Furthermore, time-activity curves were generated by applying the tumor VOI to the full dynamic data set. As described previously^38^, the dynamic parameters TTP and slope were determined. TBR_mean_, TBR_max_, TTP, and slope are considered as standard FET PET parameters throughout the manuscript.

Tumor masks were created for textural feature analysis by setting all voxels outside the tumor VOI in the summed image from 20–40 min to zero while leaving the original SUV values inside the VOI unchanged. The ECAT7 images were converted to NIFTI file format for further processing using PMOD (Version 3.5.5, PMOD Technologies Ltd.). The software LIFEx (Version 2.2, lifexsoft.org)^39^ was used for calculation of 33 textural parameters that differ in the way they are mathematically calculated. A detailed description of each textural parameter is available in the technical appendix of the LIFEx software^32^.

Finally, in order to further increase diagnostic accuracy, the standard parameters TBR_mean_, TBR_max_, TTP, and slope were combined with each other and with the textural parameters using linear logistic regression. Importantly, a maximum number of two parameters was accepted for model generation in order to limit model complexity and avoid overfitting of the data. The models were validated using cross-validation (MATLAB, R2017b. Mathworks, Inc., MA, USA). Two commonly used cross-validation methods (5-fold and 10-fold cross-validation) were applied to the best model of each patient cohort.

Due to the different spatial resolution of the two PET scanners used, the applied data correction methods and reconstruction algorithms that might directly influence the results of textural feature analyses, imaging data of each scanner type were analyzed separately. However, to evaluate robustness of parameters with respect to the different PET scanners, the complete patient cohort was also analyzed.

### Statistical Analysis

Quantitative variables are expressed as mean and standard deviation. For comparison of two different groups, the Student t-test for independent samples was used. In case of variables that were not distributed normally, the Mann-Whitney rank sum test was used. The diagnostic accuracy for IDH genotype prediction by FET PET standard parameters and textural features was evaluated by analyses of receiver-operating characteristic (ROC) curves using neuropathological results of IDH status as reference. Decision cut-off was considered optimal, when the diagnostic accuracy reached its maximum. Furthermore, the area under the ROC curve (AUC), its corresponding standard error, the 95% confidence interval, and the level of significance were evaluated to assess the diagnostic quality. As mentioned above, the standard parameters TBR_mean_, TBR_max_, TTP, and slope were combined with each other and with the textural parameters using linear logistic regression in order to further increase the accuracy of predicting the IDH genotype. The diagnostic performance of the parameter combinations was evaluated by Fisher´s exact test for 2 × 2 contingency tables. A p-value of <0.05 was considered as significant. The significance level was adjusted for multiple comparisons using the Bonferroni post-hoc correction. Statistical analyses were performed using Microsoft Excel (Excel:Mac 2011, Version 14.6, Microsoft Corp.), Mathematica (Version 10.3, Wolfram Research) and IBM SPSS Statistics (IBM Corp. Released 2012, Version 21.0, IBM Corp.).

## Electronic supplementary material

Supplementary Tables
