# Supplementary material for: Predicting IDH genotype in gliomas using FET PET radiomics
Source: Sci Rep. 2018 Sep 6;8:13328. doi: 10.1038/s41598-018-31806-7 (PMC6127131; doi:10.1038/s41598-018-31806-7)
Supplement: Supplementary file 1 — Supplementary Tables [file 41598_2018_31806_MOESM1_ESM.pdf]

SUPPLEMENTARY MATERIAL

Predicting IDH genotype in gliomas using FET PET radiomics

Scientific Reports

*Philipp Lohmann[1,2], Christoph Lerche, Elena K. Bauer, Jan Steger, Gabriele Stoffels, Tobias Blau, Veronika Dunkl, Martin Kocher, Shivakumar Viswanathan, Christian P. Filss, Carina Stegmayr, Maximillian I. Ruge, Bernd Neumaier, Nadim J. Shah, Gereon R. Fink, Karl-Josef Langen, and Norbert Galldiks

*corresponding author
[1]Inst. of Neuroscience and Medicine (INM-4), Forschungszentrum Juelich, Juelich, Germany
[2]Dept. of Stereotaxy and Functional Neurosurgery, University of Cologne, Cologne, Germany

Supplementary Table 1: Results of ROC analysis of all patients (84 patients; 26 IDH mut and 58 IDH wt gliomas)

Textural Feature	Cut-off	Accuracy	AUC	SE	95% CI	Sens.	Spec.	p
Slope [SUV/h]	0.30	0.80	0.79	0.05	0.69-0.89	0.58	0.90	< 0.01
TBR_{mean}	1.68	0.73	0.66	0.07	0.53-0.79	0.12	1.00	0.02
TTP [min]	45.00	0.73	0.75	0.05	0.64-0.85	0.27	0.93	< 0.01
LRHGE	1583.35	0.71	0.52	0.07	0.39-0.65	0.08	1.00	0.75
SkewnessH	1.59	0.71	0.53	0.07	0.39-0.67	0.31	0.90	0.66
TBR_{max}	2.07	0.71	0.59	0.07	0.45-0.73	0.08	1.00	0.20
SRHGE	987.65	0.70	0.56	0.07	0.42-0.69	0.04	1.00	0.40
HGRE	1072.25	0.70	0.59	0.07	0.46-0.72	0.08	0.98	0.17
LRE	1.35	0.70	0.51	0.07	0.38-0.64	0.04	1.00	0.92
Homogeneity_GLCM	0.18	0.70	0.52	0.07	0.39-0.65	0.04	1.00	0.74
KurtosisH	5.53	0.70	0.54	0.07	0.40-0.68	0.31	0.88	0.55
LZHGE	71800.80	0.69	0.52	0.07	0.39-0.65	0.08	0.97	0.75
SZHGE	644.05	0.69	0.56	0.07	0.42-0.69	0.08	0.97	0.43
HGZE	1048.30	0.69	0.60	0.06	0.48-0.73	0.04	0.98	0.14
EnergyH	0.05	0.69	0.53	0.07	0.39-0.68	0.23	0.90	0.64
EntropyH	1.40	0.69	0.54	0.07	0.40-0.68	0.08	0.97	0.57
Volume [mL]	2.20	0.69	0.55	0.07	0.41-0.68	0.12	0.95	0.49
ZP	0.28	0.68	0.51	0.07	0.38-0.64	0.00	0.98	0.93
ZLNU	4769.90	0.68	0.52	0.06	0.39-0.64	0.00	0.98	0.81
GLNUz	237.45	0.68	0.52	0.07	0.39-0.65	0.04	0.97	0.78
LZE	203.20	0.68	0.50	0.07	0.37-0.63	0.00	0.98	0.99
SZE	0.56	0.68	0.51	0.07	0.38-0.64	0.00	0.98	0.87
Busyness_NGLDM	5.50E+16	0.68	0.52	0.07	0.39-0.64	0.00	0.98	0.80
Contrast_NGLDM	0.17	0.68	0.54	0.07	0.41-0.67	0.00	0.98	0.53
Coarseness_NGLDM	0.02	0.68	0.50	0.07	0.37-0.63	0.00	0.98	0.99
RP	0.91	0.68	0.51	0.07	0.38-0.64	0.00	0.98	0.89
RLNU	21809.05	0.68	0.51	0.07	0.39-0.64	0.00	0.98	0.86

GLNUr	903.95	0.68	0.52	0.07	0.39-0.65	0.00	0.98	0.78
SRE	0.94	0.68	0.51	0.07	0.38-0.64	0.00	0.98	0.92
Dissimilarity_GLCM	3.82	0.68	0.55	0.07	0.42-0.68	0.00	0.98	0.50
Entropy_GLCM	2.33	0.68	0.52	0.06	0.40-0.64	0.00	0.98	0.81
Correlation_GLCM	0.89	0.68	0.56	0.07	0.43-0.69	0.00	0.98	0.41
Contrast_GLCM	25.62	0.68	0.55	0.07	0.42-0.68	0.00	0.98	0.46
Energy_GLCM	0.01	0.68	0.51	0.06	0.39-0.63	0.00	0.98	0.89
Compacity	7.95	0.68	0.52	0.07	0.39-0.65	0.00	0.98	0.76
Sphericity	0.81	0.68	0.58	0.07	0.45-0.70	0.00	0.98	0.27
LRLGE	0.002	0.52	0.51	0.07	0.92-0.37	0.46	0.55	0.92

AUC: area under the ROC curve; **CI**: confidence interval; **EnergyH**: Energy of histogram; **EntropyH**: Entropy of histogram; **GLCM**: Grey-level co-occurrence matrix; **GLNUr**: Grey-level non-uniformity for run; **GLNUz**: Grey-level non-uniformity for zone; **HGRE**: High grey-level run emphasis; **HGZE**: High grey-level zone emphasis; **KurtosisH**: Kurtosis of histogram; **LRE**: Long-run emphasis; **LRHGE**: Long-run high grey-level emphasis; **LRLGE**: Long-run low grey-level emphasis; **LZE**: Long-zone emphasis; **LZHGE**: Long-zone high grey-level emphasis; **NGLDM**: Neighbourhood grey-level different matrix; **RLNU**: Run length non-uniformity; **RP**: Run percentage; **SE**: standard error; **SkewnessH**: Skewness of histogram; **SRE**: Short-run emphasis; **SRHGE**: Short-run high grey-level emphasis; **SZE**: Short-zone emphasis; **SZHGE**: Short-zone high grey-level emphasis; **TBR**: tumor-to-brain ratio; **TTP**: time to peak; **ZLNU**: Zone length non-uniformity; **ZP**: Zone percentage

Supplementary Table 2: Results of ROC analysis of subgroup I (stand-alone PET scanner; 56 patients; 15 IDH mut and 41 IDH wt gliomas)

Feature	Cut-off	Accuracy	AUC	SE	95% CI	Sens.	Spec.	p
Slope [SUV/h]	0.30	0.80	0.74	0.07	0.59-0.88	0.53	0.90	0.01
TTP [min]	42.50	0.80	0.80	0.06	0.68-0.92	0.40	0.95	< 0.01
TBR_{mean}	2.92	0.77	0.50	0.09	0.32-.69	0.13	1.00	0.97
Volume [mL]	94.30	0.77	0.59	0.08	0.42-0.75	0.13	1.00	0.33
ZP	0.37	0.75	0.61	0.08	0.44-0.77	0.13	0.98	0.23
SZHGE	658.90	0.75	0.65	0.08	0.49-0.82	0.13	0.98	0.09
LZE	31.10	0.75	0.58	0.09	0.41-0.75	0.13	0.98	0.35
SZE	0.61	0.75	0.61	0.08	0.45-0.78	0.07	1.00	0.20
Contrast_NGLDM	0.22	0.75	0.67	0.08	0.51-0.83	0.07	1.00	0.05
RLNU	8356.75	0.75	0.58	0.08	0.42-0.75	0.07	1.00	0.35
GLNUr	389.45	0.75	0.57	0.09	0.40-0.73	0.07	1.00	0.46
LRHGE	1574.95	0.75	0.57	0.09	0.40-0.74	0.07	1.00	0.42
Dissimilarity_GLCM	4.72	0.75	0.66	0.08	0.50-0.82	0.13	0.98	0.07
Correlation_GLCM	0.82	0.75	0.67	0.08	0.51-0.83	0.13	0.98	0.05
Contrast_GLCM	34.87	0.75	0.67	0.08	0.51-0.83	0.07	1.00	0.05
Homogeneity_GLCM	0.31	0.75	0.60	0.82	0.44-0.76	0.13	0.98	0.26
Compacity	5.55	0.75	0.56	0.09	0.39-0.73	0.07	1.00	0.50
KurtosisH	2.24	0.75	0.58	0.09	0.40-0.77	0.20	0.95	0.35
SkewnessH	0.56	0.75	0.58	0.09	0.39-0.76	0.27	0.93	0.38
TBR_{max}	6.17	0.75	0.51	0.09	0.33-0.69	0.13	0.98	0.91
LZHGE	34357.35	0.73	0.57	0.09	0.40-0.74	0.13	0.95	0.44
HGZE	1048.30	0.73	0.57	0.09	0.40-0.74	0.07	0.98	0.40
Busyness_NGLDM	3.29E+15	0.73	0.61	0.78	0.45-0.76	0.13	0.95	0.23
RP	0.92	0.73	0.59	0.09	0.42-0.75	0.07	0.98	0.34
HGRE	1072.25	0.73	0.50	0.09	0.33-0.68	0.07	0.98	0.98
LRE	1.25	0.73	0.58	0.09	0.41-0.75	0.13	0.95	0.37

SRE	0.95	0.73	0.59	0.08	0.42-0.75	0.07	0.98	0.32
ZLNU	1211.90	0.71	0.55	0.08	0.39-0.71	0.13	0.93	0.59
GLNUz	139.85	0.71	0.55	0.08	0.39-0.71	0.07	0.95	0.57
Coarseness_NGLDM	0.02	0.71	0.51	0.09	0.35-0.68	0.00	0.98	0.90
SRHGE	1001.30	0.71	0.55	0.08	0.38-0.71	0.00	0.98	0.60
Entropy_GLCM	3.02	0.71	0.55	0.08	0.39-0.70	0.00	0.98	0.60
Energy_GLCM	0.01	0.71	0.50	0.08	0.35-0.66	0.00	0.98	0.97
Sphericity	0.81	0.71	0.51	0.08	0.35-0.68	0.00	0.98	0.88
EnergyH	0.08	0.71	0.56	0.09	0.38-0.74	0.00	0.98	0.51
EntropyH	1.26	0.71	0.56	0.09	0.38-0.75	0.00	0.98	0.49
LRLGE	0.002	0.63	0.60	0.09	0.43-0.77	0.53	0.66	0.28

AUC: area under the ROC curve; **CI**: confidence interval; **EnergyH**: Energy of histogram; **EntropyH**: Entropy of histogram; **GLCM**: Grey-level co-occurrence matrix; **GLNUr**: Grey-level non-uniformity for run; **GLNUz**: Grey-level non-uniformity for zone; **HGRE**: High grey-level run emphasis; **HGZE**: High grey-level zone emphasis; **KurtosisH**: Kurtosis of histogram; **LRE**: Long-run emphasis; **LRHGE**: Long-run high grey-level emphasis; **LRLGE**: Long-run low grey-level emphasis; **LZE**: Long-zone emphasis; **LZHGE**: Long-zone high grey-level emphasis; **NGLDM**: Neighbourhood grey-level different matrix; **RLNU**: Run length non-uniformity; **RP**: Run percentage; **SE**: standard error; **SkewnessH**: Skewness of histogram; **SRE**: Short-run emphasis; **SRHGE**: Short-run high grey-level emphasis; **SZE**: Short-zone emphasis; **SZHGE**: Short-zone high grey-level emphasis; **TBR**: tumor-to-brain ratio; **TTP**: time to peak; **ZLNU**: Zone length non-uniformity; **ZP**: Zone percentage

Supplementary Table 3: Results of ROC analysis of subgroup II (hybrid PET/MR scanner; 28 patients; 11 IDH mut, and 17 IDH wt gliomas)

Feature	Cut-off	Accuracy	AUC	SE	95% CI	Sens.	Spec.	p
Slope [SUV/h]	0.214	0.79	0.85	0.07	0.71-0.99	0.73	0.82	< 0.01
TBR_{mean}	2.08	0.79	0.84	0.07	0.69-0.99	0.91	0.71	< 0.01
KurtosisH	3.56	0.79	0.78	0.09	0.60-0.96	0.82	0.76	0.01
HGRE	1130.35	0.75	0.77	0.09	0.60-0.95	0.82	0.71	0.02
EnergyH	0.05	0.75	0.69	0.11	0.47-0.90	0.36	1.00	0.10
SkewnessH	1.51	0.75	0.73	0.10	0.53-0.93	0.36	1.00	0.05
TBR_{max}	3.36	0.75	0.72	0.10	0.52-0.92	0.73	0.76	0.05
ZP	0.58	0.71	0.70	0.10	0.50-0.90	0.55	0.82	0.08
Dissimilarity_GLCM	6.99	0.71	0.67	0.10	0.47-0.88	0.55	0.82	0.13
Correlation_GLCM	0.66	0.71	0.69	0.10	0.49-0.89	0.55	0.82	0.10
Contrast_GLCM	83.95	0.71	0.67	0.10	0.47-0.88	0.55	0.82	0.13
Compacity	4.09	0.71	0.67	0.11	0.47-0.88	0.64	0.76	0.13
EntropyH	1.48	0.71	0.72	0.10	0.52-0.91	0.36	0.94	0.06
ZLNU	757.85	0.68	0.66	0.11	0.45-0.86	0.55	0.76	0.17
GLNUz	80.10	0.68	0.65	0.11	0.44-0.86	0.64	0.71	0.20
LZHGE	12922.60	0.68	0.63	0.11	0.42-0.85	0.64	0.71	0.25
SZHGE	797.85	0.68	0.61	0.11	0.40-0.82	0.55	0.76	0.34
HGZE	1134.70	0.68	0.68	0.10	0.49-0.88	0.64	0.71	0.11
LZE	7.11	0.68	0.68	0.10	0.49-0.88	0.55	0.76	0.11
SZE	0.70	0.68	0.68	0.10	0.48-0.88	0.55	0.76	0.11
Contrast_NGLDM	0.36	0.68	0.68	0.11	0.48-0.88	0.64	0.71	0.12
RP	0.95	0.68	0.66	0.11	0.45-0.87	0.55	0.76	0.17
RLNU	2070.30	0.68	0.69	0.10	0.49-0.89	0.45	0.82	0.10
GLNUr	166.95	0.68	0.67	0.10	0.47-0.87	0.64	0.71	0.14
LRHGE	1342.55	0.68	0.62	0.11	0.40-0.83	0.64	0.71	0.31
LRLGE	0.002	0.68	0.67	0.11	0.46-0.88	0.64	0.71	0.13

SRHGE	1057.80	0.68	0.60	0.12	0.37-0.83	0.27	0.94	0.38
LRE	1.15	0.68	0.65	0.11	0.43-0.86	0.55	0.76	0.20
SRE	0.97	0.68	0.68	0.10	0.48-0.88	0.55	0.76	0.12
Homogeneity_GLCM	0.24	0.68	0.64	0.11	0.43-0.85	0.55	0.76	0.22
Volume [mL]	4.50	0.68	0.69	0.10	0.49-0.89	0.45	0.82	0.10
TTP [min]	25.00	0.64	0.64	0.11	0.43-0.85	0.91	0.53	0.21
Coarseness_NGLDM	0.002	0.64	0.64	0.11	0.43-0.85	0.73	0.59	0.21
Sphericity	0.87	0.61	0.64	0.11	0.44-0.85	0.09	1.00	0.20
Busyness_NGLDM	1.71E+15	0.61	0.57	0.11	0.36-0.79	0.82	0.47	0.53
Entropy_GLCM	2.80	0.61	0.57	0.11	0.36-0.79	0.27	0.82	0.53
Energy_GLCM	0.004	0.57	0.50	0.11	0.29-0.72	0.00	0.94	0.98

AUC: area under the ROC curve; **CI**: confidence interval; **EnergyH**: Energy of histogram; **EntropyH**: Entropy of histogram; **GLCM**: Grey-level co-occurrence matrix; **GLNUr**: Grey-level non-uniformity for run; **GLNUz**: Grey-level non-uniformity for zone; **HGRE**: High grey-level run emphasis; **HGZE**: High grey-level zone emphasis; **KurtosisH**: Kurtosis of histogram; **LRE**: Long-run emphasis; **LRHGE**: Long-run high grey-level emphasis; **LRLGE**: Long-run low grey-level emphasis; **LZE**: Long-zone emphasis; **LZHGE**: Long-zone high grey-level emphasis; **NGLDM**: Neighbourhood grey-level different matrix; **RLNU**: Run length non-uniformity; **RP**: Run percentage; **SE**: standard error; **SkewnessH**: Skewness of histogram; **SRE**: Short-run emphasis; **SRHGE**: Short-run high grey-level emphasis; **SZE**: Short-zone emphasis; **SZHGE**: Short-zone high grey-level emphasis; **TBR**: tumor-to-brain ratio; **TTP**: time to peak; **ZLNU**: Zone length non-uniformity; **ZP**: Zone percentage

Supplementary Table 4: Results of best parameter combinations

Patient cohort	Parameter 1	Parameter 2	Accuracy	Δ[a]	Sens.	Δ[b]	Spec.	Δ[c]	p
Complete (n = 84)	Slope [SUV/h]	SZHGE	0.81	+ 1%	0.54	- 4%	0.93	- 1%	< 0.01
Subgroup I (n = 56)	TTP [min]	SZHGE	0.84	+ 4%	0.53	+ 13%	0.95	± 0%	< 0.01
	Slope [SUV/h]	Contrast_GLCM	0.82	+ 2%	0.47	- 6%	0.95	+ 5%	< 0.01
	TBR_{mean}	TTP [min]	0.82	+ 5%	0.47	+ 34%	0.95	- 5%	< 0.01
	TBR_{max}	TTP [min]	0.82	+ 7%	0.47	+ 34%	0.95	- 3%	< 0.01
Subgroup II (n = 28)	TBR_{mean}	SZHGE	0.93	+ 14%	0.91	± 0%	0.94	+ 23%	< 0.01
	TBR_{mean}	Slope [SUV/h]	0.89	+ 10%	0.91	± 0%	0.88	+ 17%	< 0.01
	Slope [SUV/h]	SZE	0.86	+ 7%	0.91	+ 18%	0.82	± 0%	< 0.01
	Slope [SUV/h]	Contrast_NGLDM	0.86	+ 7%	0.91	+ 18%	0.82	± 0%	< 0.01
	Slope [SUV/h]	RLNU	0.86	+ 7%	0.91	+ 18%	0.82	± 0%	< 0.01
	Slope [SUV/h]	GLNUr	0.86	+ 7%	0.91	+ 18%	0.82	± 0%	< 0.01
	Slope [SUV/h]	Sphericity	0.86	+ 7%	0.82	+ 9%	0.88	± 0%	< 0.01
	Slope [SUV/h]	Volume [mL]	0.86	+ 7%	0.91	+ 18%	0.82	± 0%	< 0.01
	Slope [SUV/h]	TTP [min]	0.86	+ 7%	0.73	± 0%	0.94	+ 12%	< 0.01
	TBR_{mean}	ZP	0.86	+ 7%	0.82	- 9%	0.88	+ 17%	< 0.01
	TBR_{mean}	SZE	0.86	+ 7%	0.91	± 0%	0.88	+ 17%	< 0.01
	TBR_{mean}	Contrast_NGLDM	0.86	+ 7%	0.82	- 9%	0.82	+ 11%	< 0.01
	TBR_{mean}	RP	0.86	+ 7%	0.82	- 9%	0.88	+ 17%	< 0.01
	TBR_{mean}	LRHGE	0.86	+ 7%	0.82	- 9%	0.88	+ 17%	< 0.01
	TBR_{mean}	SRE	0.86	+ 7%	0.91	± 0%	0.88	+ 17%	< 0.01
	TBR_{mean}	Dissimilarity_GLCM	0.86	+ 7%	0.91	± 0%	0.82	+ 11%	< 0.01
	TBR_{mean}	Correlation_GLCM	0.86	+ 7%	0.91	± 0%	0.82	+ 11%	< 0.01
	TBR_{mean}	Contrast_GLCM	0.86	+ 7%	0.82	- 9%	0.82	+ 11%	< 0.01

TBR_{mean}	Homogeneity_GLCM	0.86	+ 7%	0.55	- 36%	0.88	+ 17%	< 0.01
SRHGE	RLNU	0.82	+ 14%	0.64	+ 37%	1.00	+ 6%	< 0.01
HGRE	ZLNU	0.82	+ 7%	0.64	+ 18%	0.94	+ 23%	< 0.01
HGRE	RLNU	0.82	+ 7%	0.73	+ 9%	0.94	+ 23%	< 0.01
SkewnessH	GLNUr	0.82	+ 7%	0.55	+ 19%	0.88	- 12%	< 0.01
Volume [mL]	SRHGE	0.82	+ 14%	0.64	+ 19%	1.00	+ 18%	< 0.01
Volume [mL]	HGRE	0.82	+ 14%	0.82	+ 37%	0.94	+ 12%	< 0.01
Slope [SUV/h]	ZP	0.82	+ 3%	0.91	+ 18%	0.82	± 0%	< 0.01
Slope [SUV/h]	SZHGE	0.82	+ 3%	0.91	+ 18%	0.76	- 6%	< 0.01
Slope [SUV/h]	LZE	0.82	+ 3%	0.91	+ 18%	0.76	- 6%	< 0.01
Slope [SUV/h]	RP	0.82	+ 3%	0.82	+ 9%	0.76	- 6%	< 0.01
Slope [SUV/h]	LRLGE	0.82	+ 3%	0.91	+ 18%	0.82	± 0%	< 0.01
Slope [SUV/h]	SRE	0.82	+ 3%	0.82	+ 9%	0.76	- 6%	< 0.01
Slope [SUV/h]	Dissimilarity_GLCM	0.82	+ 3%	0.82	+ 9%	0.82	± 0%	< 0.01
Slope [SUV/h]	Homogeneity_GLCM	0.82	+ 3%	0.73	± 0%	0.82	± 0%	< 0.01
Slope [SUV/h]	Compacity	0.82	+ 3%	0.73	± 0%	0.88	+ 6%	< 0.01
TBR_{mean}	SRHGE	0.82	+ 3%	0.73	- 18%	0.88	+ 17%	< 0.01
TBR_{mean}	LRE	0.82	+ 3%	0.73	- 18%	0.88	+ 17%	< 0.01

GLCM: Grey-level co-occurrence matrix; **GLNUr**: Grey-level non-uniformity for run; **HGRE**: High grey-level run emphasis; **LRE**: Long-run emphasis; **LRHGE**: Long-run high grey-level emphasis; **LRLGE**: Long-run low grey-level emphasis; **LZE**: Long-zone emphasis; **NGLDM**: Neighborhood grey-level different matrix; **RLNU**: Run length non-uniformity; **RP**: Run percentage; **SkewnessH**: Skewness of histogram; **SRE**: Short-run emphasis; **SRHGE**: Short-run high grey-level emphasis; **SZE**: Short-zone emphasis; **SZHGE**: Short-zone high grey-level emphasis; **TBR**: tumor-to-brain ratio; **TTP**: time to peak; **ZLNU**: Zone length non-uniformity; **ZP**: Zone percentage

[a] Relative difference to accuracy of respective single parameter (Parameter 1)

[b] Relative difference to sensitivity of respective single parameter (Parameter 1)

[c] Relative difference to specificity of respective single parameter (Parameter 1)
